# Expression of HIV-1 antigens in plants as potential subunit vaccines

**DOI:** 10.1186/1472-6750-8-53

**Published:** 2008-06-23

**Authors:** Ann Meyers, Ereck Chakauya, Enid Shephard, Fiona L Tanzer, James Maclean, Alisson Lynch, Anna-Lise Williamson, Edward P Rybicki

**Affiliations:** 1 Institute of Infectious Disease and Molecular Medicine, Faculty of Health Sciences, University of Cape Town, Observatory 7925, South Africa; 2 Department of Molecular and Cell Biology, Faculty of Science, University of Cape Town, P. Bag X3 Rondebosch 7701, South Africa; 3 CSIR Biosciences, Pretoria 0001, South Africa; 4 MRC/UCT Liver Research Centre, Department of Medicine, Faculty of Health Sciences, University of Cape Town, Observatory 7925, South Africa; 5 National Health Laboratory Service, Groote Schuur Hospital, Observatory 7925, South Africa

## Abstract

**Background:**

Human immunodeficiency virus type 1 (HIV-1) has infected more than 40 million people worldwide, mainly in sub-Saharan Africa. The high prevalence of HIV-1 subtype C in southern Africa necessitates the development of cheap, effective vaccines. One means of production is the use of plants, for which a number of different techniques have been successfully developed. HIV-1 Pr55Gag is a promising HIV-1 vaccine candidate: we compared the expression of this and a truncated Gag (p17/p24) and the p24 capsid subunit in *Nicotiana *spp. using transgenic plants and transient expression via *Agrobacterium tumefaciens *and recombinant tobamovirus vectors. We also investigated the influence of subcellular localisation of recombinant protein to the chloroplast and the endoplasmic reticulum (ER) on protein yield. We partially purified a selected vaccine candidate and tested its stimulation of a humoral and cellular immune response in mice.

**Results:**

Both transient and transgenic expression of the HIV antigens were successful, although expression of Pr55Gag was low in all systems; however, the *Agrobacterium*-mediated transient expression of p24 and p17/p24 yielded best, to more than 1 mg p24/kg fresh weight. Chloroplast targeted protein levels were highest in transient and transgenic expression of p24 and p17/p24. The transiently-expressed p17/p24 was not immunogenic in mice as a homologous vaccine, but it significantly boosted a humoral and T cell immune response primed by a *gag *DNA vaccine, pTHGagC.

**Conclusion:**

Transient agroinfiltration was best for expression of all of the recombinant proteins tested, and p24 and p17/p24 were expressed at much higher levels than Pr55Gag. Our results highlight the usefulness of plastid signal peptides in enhancing the production of recombinant proteins meant for use as vaccines. The p17/p24 protein effectively boosted T cell and humoral responses in mice primed by the DNA vaccine pTHGagC, showing that this plant-produced protein has potential for use as a vaccine.

## Background

The acquired immunodeficiency syndrome (AIDS) resulting from infection by human immunodeficiency virus type 1 (HIV-1) is arguably the greatest medical and scientific challenge to face humankind in the past decade. More than 40 million people worldwide live with the virus, and 5 million were infected in 2004 alone. Two thirds of all HIV-1 infections occur in sub-Saharan Africa and the epidemic is still spreading at unprecedented rates [1].

It has been proposed that the only means of halting this epidemic will be the introduction of a large-scale immunisation programme, preferably using cheaply-produced vaccines. There are numerous vaccine initiatives currently in progress globally [2], but the number of different subtypes of HIV-1 prevalent in different geographic areas has complicated the prospects for a universal HIV-1 vaccine. HIV-1 subtype C is the predominant HIV in southern Africa; therefore, South African isolates of this subtype have been used in the development of candidate DNA and other virally- and bacterially-vectored and subunit vaccines for South Africa under the auspices of the South African AIDS Vaccine Initiative (SAAVI) [3-5]. Because of the extent of HIV-1 infection in southern Africa, any immunisation programme will require enormous quantities of vaccine. DNA vaccines are expensive to manufacture, as are recombinant viral and subunit vaccines produced via cell culture. This makes the proposition costly and possibly unfeasible, particularly for the poorer developing countries where HIV infection is prevalent. There is therefore a strong need to develop vaccine expression systems which have the potential to produce vaccines at low cost for these countries.

One of the cheapest possible means of producing subunit vaccines is via plants [6-9]. Expression hosts include crop plants such as *Nicotiana tabacum *(tobacco) and other *Nicotiana *spp., alfalfa, rice, wheat, potato and soybeans, amongst a number of others. Different expression systems include transient viral vector expression [10-15], *Agrobacterium tumefaciens*-mediated transient expression [16-18], and the generation of stable transgenic plants [19-21] or cultured plant cells [22] expressing the protein of interest. The majority of plant-produced recombinant proteins have been produced by transgenic plants, and in many cases, transplastomic plants [23]. However, transient expression is a viable alternative: the short production cycle coupled with codon optimisation, suitable promoters and the help of silencing suppressors such as plant virus-derived p19 and NSs [18] means that it can be scaled up to produce high protein yields in a very short time [7].

There are several advantages to using plant-produced vaccine antigens: there is proper protein folding equivalent to other eukaryotic systems, the raw material cost is lower, the process is highly scalable, large amounts can be produced at relatively low cost, multiple vaccines may be produced together and they may be expressed in edible plant organs, which would make oral delivery convenient [7,24-26]. However, poor expression levels in transgenic plants and the complex regulatory problems surrounding plant-produced vaccines are the major challenges [27]. Moreover, reports of inadvertent mixing into the food chain of pharmaceutical proteins from transgenic plants in the USA [28] have put transient plant expression into the spotlight as a *bona fide *production strategy in its own right.

**Figure 1:**
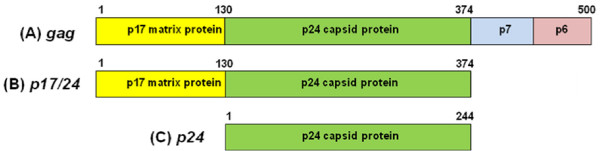
**The HIV-1 Gag-derived proteins used in this study**. Scale diagram showing (A) native Pr55Gag ORF organisation in the *gag *gene, (B) the p17/p24 fusion protein ORF, (C) p24 ORF. ORFs labelled p7, p6 represent minor proteins.

The HIV-1 Gag precursor protein Pr55 Gag as well as proteins p17 (MA or matrix protein) and p24 (CA or capsid protein) resulting from cleavage of Pr55Gag by the viral protease, are potentially good candidates for the development of HIV subunit vaccines (see Figure 1). Pr55Gag expressed in a variety of cell systems can assemble and bud through the plasma membrane to form highly immunogenic virus-like particles (VLPs) [29,30]. These VLPs are non-infectious and morphologically similar to immature HIV particles, stimulate potent cellular and humoral responses, and are safe to administer to animals and humans [31]. Gag VLPs are a promising vaccine candidate because Gag contains the highest density of cytotoxic T-lymphocyte (CTL) epitopes of any HIV protein [32,33], and cellular immune responses to Gag in infected people are correlated with control of infections; they are highly immunogenic and elicit Th1-biased responses in experimental animals at low dosage levels [30]; they are relatively stable, and they can easily be used as carriers for other peptides [29]. The native p17 facilitates the intra-membrane associations necessary for viral assembly and release [34,35] as well as being involved in the transport of the viral pre-integration complex into the nucleus [36]. It is well-documented that the risk of AIDS is greatly increased in individuals with falling titres of p24 antibodies [37-40], suggesting that high anti-p24 antibody titres might be necessary to maintain a disease-free state. It is also significant that p17 and p24 together contain the highest density of cytotoxic T-lymphocyte (CTL) epitopes of the HIV-1 proteome [33]. There are also indications that p17/24 fusion proteins may form VLPs or tubular structures [41], which could make them a good carrier for immunogenic presentation of foreign epitopes.

Studies done on plant-expressed HIV-1 vaccines have largely focused on HIV-1 Env epitopes fused to virus particles or other proteins, to produce chimaeric virus particles which have been tested for their ability to produce an appropriate antibody response [42-46]. A few studies have also been carried out using the Tat protein [47,48]. Zhang et al. [49,50] showed that HIV-1 p24 could be expressed as a fusion protein with tomato bushy stunt virus (TBSV) capsid protein in tomatoes. However, the inserted *p24 *open reading frame (ORF) was not stably maintained. The same researchers subsequently generated stable transgenic *N. tabacum *expressing the same HIV-1 p24 protein at levels of 0.35% of total soluble protein (TSP) [26]. More recently, Pérez-Filgueira [50] et al. showed that a recombinant tobacco mosaic virus (TMV)-derived vector expressing subtype C HIV-1 p24 in *N. benthamiana *yielded levels of 100 mg per kg fresh leaf weight. Furthermore, immunisation of rabbits using purified recombinant p24 induced a humoral response in inoculated rabbits that was strong and specific to p24. However, there are no studies reported to date on the expression of HIV-1 Pr55Gag or p17 in plants, and HIV-1 p24 is not seen by many as a good vaccine candidate. While its expression in plants has not been investigated, recombinant HIV-1 p17/24 fusion protein has successfully been produced in insect cells via recombinant baculovirus [41] for mapping domains involved in VLP formation.

We have previously expressed HIV-1 subtype C isolate Du422 Pr55Gag via recombinant baculovirus in insect cells, and shown that it assembled and budded to form VLPs, and that these were highly immunogenic in mice [30]. In this study we explored and compared the use of transient and transgenic plant expression systems for the production of recombinant HIV-1 subunit proteins p24 and p24/p17 as well as of Pr55Gag and possibly VLPs. We investigated the usefulness of codon optimisation and of targeting recombinant proteins to the ER and chloroplasts as a means of improving yields, and investigated whether transient expression via recombinant tobamoviruses or *Agrobacterium tumefaciens *produced better yields than the *Agrobacterium*-engineered transgenic system. Finally, we assessed both the cellular and humoral immune responses to HIV-1 p17/p24 (one of the most highly-expressed plant-produced HIV proteins) in mice inoculated with this protein, in order to assess the viability of a plant-produced HIV-1 vaccine.

## Results

### TMV vector expression

Expressed protein levels measured as μg p24/kg fresh leaf weight using the TMV vector expression system are shown in Table 1. Expression of full length Pr55Gag from the wt*gag *gene was almost undetectable (<0.01 μg p24/kg fresh leaf wt) compared with *Nicotiana *spp. codon-optimised n*gag*-expressed Pr55Gag levels, which ranged from 0.46 to 2.0 μg p24/kg fresh leaf wt. Expressed protein levels from all three versions of the *p24 *gene (*Nicotiana *spp.- and human codon-optimised and wild type) were significantly higher (100 to 10000 times greater) than the p24 levels obtained with *gag*, ranging from 167 to 17350 μg p24/kg fresh leaf wt. Although plant-optimised n*p24 *expression levels were the highest, with wt*p24 *expressed protein levels being about 1.2 times and h*p24 *expressed protein levels being at least 10 times less, p24 levels varied considerably within the specific version tested. Despite the low levels of recombinant protein expression, samples of crude plant sap from *N. benthamiana *infected with the pBSGngag recombinant TMV RNA that were immunotrapped with HIV-1 p17 antiserum and viewed under the electron microscope, showed the presence of VLPs approximately 110 to 120 nm in diameter (data not shown). These were very similar in shape and size to baculovirus-produced Pr55Gag VLPs [30]. Plants infected with rTMV expressing p24 did not produce any particles (not shown). Given the very low yield of the Pr55Gag protein, however, these preliminary results were not followed up.

**Table 1:** Yields of recombinant proteins obtained in *Nicotiana *spp. using the TMV expression system.

**Recombinant protein (non-targeted)**	**TMV Expression (μg p24/kg)**
**Full length Pr55Gag**	
wt*gag*	<0.01
n*gag*	0.46 – 2.0
	
**p*24***	
wt*p24*	172 – 13900
n*p24*	230 – 17350
h*p24*	167 – 1000

Levels of protein are expressed as μg p24 per kg of fresh leaf weight.

wt = wildtype codon usage; n = *Nicotiana *spp. codon-optimised; h = human codon-optimised

### *A. tumefaciens*-mediated transient expression

To investigate the possibility of expressing HIV-1 p24, p17/24 and Pr55Gag in plants for vaccine purposes, three sets of constructs encoding cytosol-retained, ER-targeted or chloroplast-targeted proteins (Table 2), were generated and agroinfiltrated into *N. benthamiana *plants. In addition, the effects of myristylation of p17/24 were investigated, as this is known to affect the accumulation of Pr55Gag in cells.

**Table 2:** Summary of the recombinant constructs used for *Agrobacterium*-mediated transient and transgenic expression.

**Vector**	**Subcellular Target**	**Insert**	**Clone**
pTRAc	cytosol	*p24*	pTRA-Cp24
		*p17/p24*	pTRA-Cp17/p24-M
			pTRA-Cp17/p24-NM
		*gag*	pTRA-Cgag

pTRAkc-ERH	ER	*p24*	pTRA-ERp24
		*p17/p24*	pTRA-ERp17/p24-M
			pTRA-ERp17/p24-NM
		*gag*	pTRA-ERgag

pTRAkc-rbcS1-cTP	chloroplast	*p24*	pTRA-CPTp24
		*p17/p24*	pTRA-CPTp17/p24-M
			pTRA-CPTp17/p24-NM
		*gag*	pTRA-CPTgag

All genes were *Nicotiana *spp. codon-optimised

M = myristylated; NM = non-myristylated

The expression of the recombinant proteins Pr55Gag, p24 and myristylated p17/p24 was measured using a p24 antigen ELISA. A comparison of the three transient recombinant protein expression levels in *N. benthamiama *leaves is shown in column A (Table 3). In leaf tissue that was syringe-infiltrated with constructs expressing the cytosol-retained proteins, p17/p24 was expressed at the highest level compared with expression of both p24 and Pr55Gag which were 10 times lower. In leaf tissue infiltrated with ER-targeted constructs, p24 was expressed at the highest level (ranging from 3700 to 16100 μg/kg) when compared with expression of p17/p24 (200 μg/kg) and Pr55Gag (8 to 13 μg/kg). In leaf tissue infiltrated with chloroplast-targeted constructs, both p24 and myristylated p17/p24 were expressed at similar levels (4000 μg/kg) when compared with expression of Pr55Gag (1 to 29 μg/kg). Overall, ER-targeted p24 and chloroplast-targeted p24 and p17/p24 yielded p24 levels which were greater than 1 mg/kg fresh weight. The expression of Pr55Gag was low in all of the targeted intracellular sites. No p24 antigen was detectable in plants infiltrated with buffer only as the control.

**Table 3:** Comparison of yields of recombinant HIV-1 p24, p17/24 and Gag proteins obtained in *N. benthamiana *by (A) *A. tumefaciens-*mediated transient expression or (B) transgenic expression.

	**A**	**B**
**Recombinant protein**	**Transient Expression (μg p24/kg)**	**Transgenic Expression (μg p24/kg)**

**Pr55Gag**		
GagC	7 – 44	D
GagER	8 – 13	0.13 – 7
GagCPT	1 – 29	0.03 – 48
		
**p24**		
p24C	0.01	0.04
p24ER	3691 – 16148	0.01 – 1.19
p24CPT	937 – 4014	636 – 2994
		
**p17/p24**		
p17/p24C	220	-
p17/p24ER	220	<1
p17/p24CPT	4800	5–230

Levels of protein are expressed as μg p24 per kg of fresh leaf weight. All genes were *Nicotiana *spp. codon-optimised.

C, ER and CPT in construct names indicate whether the protein remained in the cytosol, or was targeted to the endoplasmic reticulum or the chloroplast, respectively.

D: regenerants died during transplantation

-: no transgenic plants generated

**Figure 2:**
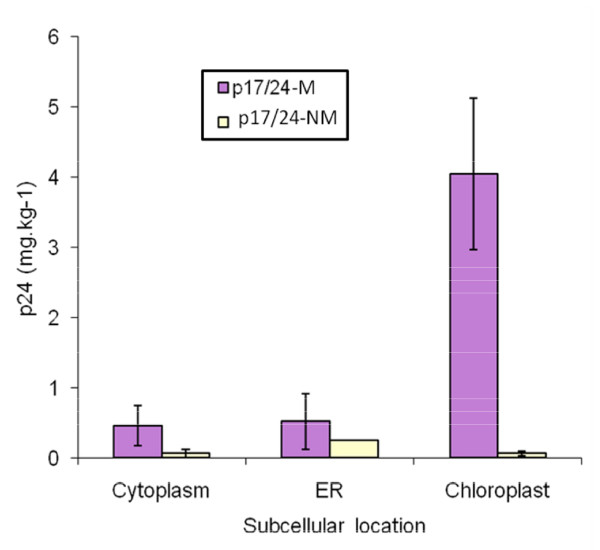
**The effect of intracellular localisation on accumulation of myristylated p17/24**. Transient expression of HIV-1 p17/24 protein in *Agrobacterium*-infiltrated *N. benthamiana *was measured by HIV-1 p24 ELISA 4 days after infiltration.

We separately investigated the effect of removing the myristylation signal on p17/p24 expression by comparing G2A-mutated p17/24 (NM) and myristylated p17/24 (M) protein expression (Figure 2). Removal of the signal reduced cytosolic protein accumulation by 9-fold and ER accumulation by only ~2 fold, but resulted in 50 to 70-fold yield reduction for the chloroplast localised protein when compared to the myristylated version, at 4 mg/kg. Protein expression for the three non-myristylated p17/24 constructs (pTRA-Cp17/p24-NM, pTRA-CPTp17/p24-NM and pTRA-ERp17/p24-NM) was highest in the ER (241 μg/kg), and about the same in cytoplasm and chloroplasts (~60 μg/kg).

**Figure 3:**
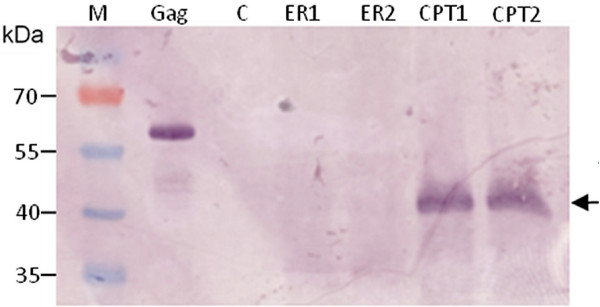
**Western blot analysis of HIV p17/24 from infiltrated *N. benthamiana *leaves and fractionation of purified protein**. Clarified sap samples were electrophoresed on a 12% SDS-PA gel and blotted onto nylon membrane. The membrane was probed with a rabbit polyclonal anti-p24. M, molecular weight marker; Gag, HIV B Gag reference sample; C, extract from leaves infiltrated with infiltration buffer; ER1 and ER2, extracts from two pTRA-ERp17/p24-M infiltrated leaves; CPT1 and CPT2, extracts from two pTRA-CPTp17/p24-M infiltrated leaves.

The western blot detection of transiently-expressed p17/p24 protein in unfractionated leaf extracts is shown in Figure 3: the product is clearly visible, as a strong single band of appropriate size, in extracts of plants where protein was targeted to the chloroplasts. In contrast, ER-targeted protein is barely visible as a band in adjacent lanes.

### HIV antigen expression in transgenic *N. tabacum*

Expressed p24 protein levels from transgenic plants are shown in column B (Table 3). No HIV-1 p24 protein was detected in non-transgenic control samples. Eight transgenic plants expressing chloroplast-targeted p24 (n*p24*C) were regenerated. The highest p24 level measured was about half of that reported for the corresponding transiently-expressed protein. Three transgenic plants expressing ER-targeted and one expressing cytoplasm-targeted p24 were regenerated. Expressed p24 levels ranged from 0.01 to 1.19 μg/kg fresh leaf wt for n*p24*ER and 0.04 μg/kg fresh leaf wt for n*p24*C.

Five putative transgenic plants were generated for pTRA-ERp17/p24-M and extracts from leaves of these plants shown to have less than 1 μg/kg of p24 expressed in a preliminary p24 ELISA screen (Table 3 column B). Eight transgenic lines were generated from pTRA-CPTp17/p24-M-producing transgenic plants. Preliminary screening showed the expression of approximately 5 to 230 μg p24/kg FW. No transgenic plants were generated from pTRA-Cp17/p24-M. No product was amplified from control plant genomic DNA.

Twenty-six transgenic plants expressing chloroplast-targeted Pr55Gag were regenerated on kanamycin selection medium. Recombinant protein levels (Table 3 column B) were similar to those obtained with the corresponding vectors in the transient expression studies which were generally low: these ranged from 0.03 to 48 μg p24/kg fresh leaf wt. Twenty-eight transgenic plants expressing ER-targeted Pr55Gag were regenerated with expressed protein levels ranging from 0.13 to 7 μg p24/kg fresh leaf wt. Although there were 8 cytoplasm-targeted Pr55Gag regenerants, these did not survive transplantation and hardening off and we were not able to measure the p24 expression levels. Overall, Pr55Gag expression was negligible compared with that of p24 and p17/p24.

### Cellular and humoral response of mice to the p17/p24 (pTRAk-CPTp17/p24-M)

Although transiently-expressed ER-targeted p24 (p24ER) on its own yielded the highest levels of p24 per kg FW (Table 3 column A), it was decided to carry out immunological studies in mice using the chloroplast-targeted p17/p24 protein (p17/p24CPT). From the point of view of developing a candidate vaccine it provides a greater number of epitopes for the induction of a broad immune response. The possibility that this protein may form VLPs or tubular structures also supports its use in immunogenicity studies [41]. Particulate p17/p24 protein is proposed to be more effective in stimulating a cellular immune response [51]. Partially-purified protein extract fractionated by SDS-PAGE contained predominantly a single polypeptide of the appropriate size (not shown).

The cellular immune response to p17/p24 partially-purified leaf protein was investigated in BALB/c mice, either alone, or in a prime boost inoculation regimen where the DNA vaccine pTHGagC was used to prime the immune response and p17/p24 protein to boost this response. Cellular responses were assayed by IFN-γ ELISPOT and antibody responses by ELISA and the specificity of the antibodies confirmed using the New LAV Blot 1 western blot kit.

**Figure 4:**
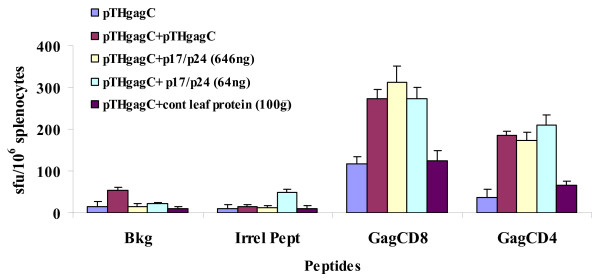
**IFN-γ ELISPOT analysis of Gag T cell responses after vaccination of BALB/c mice**. Inoculations with the indicated antigens were given as specified in the methods and all groups of mice were sacrificed on day 40. Reactions in the IFN-γ ELISPOT assay were done in triplicate with the indicated Gag peptides, an irrelevant peptide (irrel pept) or absence of peptide (Bkg) and bars are the average number of spot forming units (sfu) ± SD/10^6 ^splenocytes. Data are from a representative experiment with splenocytes pooled from 5 mice per group.

For all groups of mice the average background responses in the absence of peptide and to an irrelevant peptide was not greater than 50 ± 6 sfu/10^6 ^splenocytes (Figure 4). The strong response to a Gag CD8+ T cell peptide of 117 sfu/10^6 ^splenocytes that was achieved with a single inoculation of pTHGagC was increased 2.3 fold (p < 0.01) by a booster inoculation with 64 ng of p17/p24 protein (Figure 4). No improvement in this response was achieved when the dose was increased to 646 ng p17/p24 protein. pTHGagC induced a weak response just above background to a Gag CD4+ T cell peptide, which was significantly boosted (4.7 fold) by 64 ng p17/p24 protein to 209 sfu/10^6 ^splenocytes (p < 0.01). As seen with the response to the CD8+ T cell peptide, a higher dose of p17/p24 protein did not increase the magnitude of the immune response to the Gag CD4+ T cell peptide. These increases in cellular immune responses to Gag induced by p17/p24 protein were specific, as there was no significant change in the response to pTHGagC when control leaf protein was used as the boost (Figure 4). Interestingly, the level of response to the Gag CD8+ and CD4+ T cell peptides after a prime with pTHGagC and boost with p17/p24 protein was similar to that of two doses of pTHGagC (Figure 4). The mice in groups 6 and 7 that were inoculated with the p17/p24 protein alone showed no cellular immune response to Gag above background, 12 days after inoculation (Figure 4).

**Figure 5:**
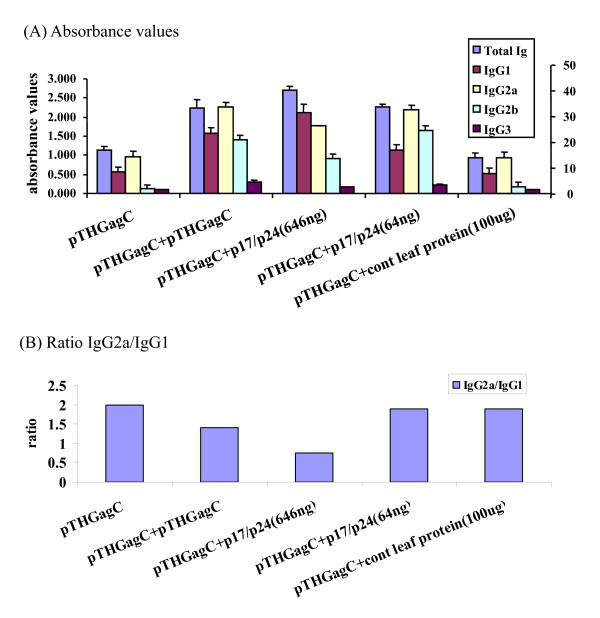
**Gag-specific serum total IgG and IgG subtypes**. Mouse groups were inoculated with the indicated antigens as specified in the methods and serum isolated on day 40 for antibody measurements by ELISA quantifying total IgG and IgG subtypes IgG1, IgG2a, IgG2b and IgG3 against Gag. Pooled serum samples from 5 mice per group were tested at 1:1000 dilution. (A) Bars represent the average absorbance ± SD for triplicate values, and represent the level of total IgG and IgG subtypes IgG1, IgG2a, IgG2b and IgG3 against Gag above pre-bleed absorbance values which were not more than 0.003. Ratio of absorbance values to prebleed values is shown on the right. (B) Ratio of IgG2a vs IgG1 for the indicated groups of mice.

The humoral arm of the specific immune response to Gag for the 8 groups of immunized mice was evaluated by looking at serum anti-Gag total IgG and IgG subtypes (Figure 5A). Significant levels (p < 0.01) of Gag-specific total IgG antibodies and IgG subtypes above those in the pre-bleed serum were obtained for groups 1 – 5 inoculated with either pTHGagC or primed with pTHGagC then boosted with p17/p24 protein (Figure 5A).

The high levels of Gag-specific total IgG in response to pTHGagC of 17 fold above background (p < 0.01) increased 40 fold (p < 0.01) and 34 fold (p < 0.01) above background when the mice were boosted with either 646 ng or 64 ng p17/p24 protein respectively (Figure 5A). These differences in the level of Gag-specific total IgG elicited by booster doses of 646 ng and 64 ng of p17/p24 protein were significant (p < 0.05) (Figure 5A). Determination of Gag-specific serum IgG subtypes indicated the presence of significant (p < 0.01) levels of IgG1, IgG2a, IgG2b and IgG3 above background pre-bleed values (Figure 5A). Boosting pTHGagC primed mice with pTHGagC or 64 ng leaf protein containing p17/p24 protein induced higher IgG2a than IgG1 levels whereas boosting with 646 ng p17/p24 protein reversed this ratio (Figure 5B). No Gag-specific antibodies above that in the pre-bleed serum were obtained for groups 6 – 8 inoculated with either dose of p17/p24 protein or control leaf protein (data not shown).

**Figure 6:**
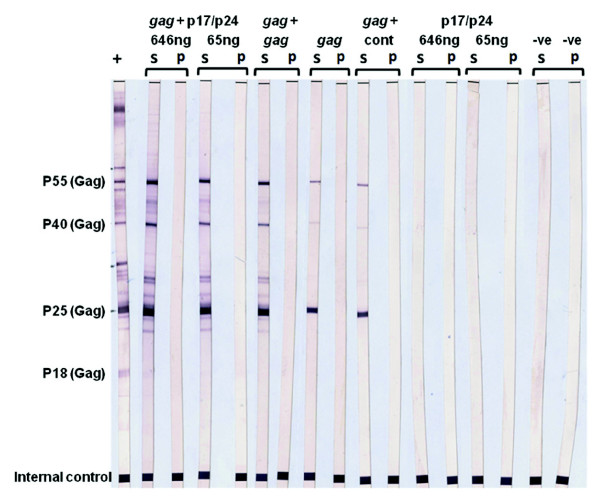
**Western blot detection of Gag antibodies in mouse serum**. The content of Gag antibody in mouse serum was detected using commercial western blot strips as described in the methods. +, positive control serum; -ve, negative control serum; S, mouse serum taken at day 40 after inoculation as indicated and described in methods; P, pre-inoculation mouse serum. The inoculation regimen for each set of strips is indicated above the strips: these were *gag *DNA prime – p17/p24 boost; *gag *prime-*gag *boost; *gag *single inoculation; p17/p24 single inoculations.

A commercial western blot system for determining HIV-1 antibodies confirmed the presence of antibodies to Gag in the serum of inoculated mice (Figure 6). In addition, judged from the intensity of the bands on the strips, the ability of p17/p24 to boost the antibody response induced by pTHGagC is clearly visible.

## Discussion

Our group has previously investigated the suitability of a baculovirus-expressed HIV-1 Pr55 Gag virus-like particle (VLP) preparation as a candidate HIV vaccine, in combination with a DNA *gag *vaccine [30]. Part of our objective in this study was to investigate the feasibility of expressing the same gene in plants via recombinant TMV as a cheaper means of making VLPs. While a plant codon-optimised version of the gene produced a Pr55Gag protein which apparently formed recognisable VLPs (data not shown), the yield of protein was extremely low. We investigated whether yield could be improved by truncation or differential localisation of the protein, and showed that sequences downstream of the p24 ORF negatively affected expression, given that either p24 or p17/24 proteins were expressed relatively well, both in transient and transgenic expression systems. Interestingly, while expression of the *gag*-derived genes used here did benefit from plant codon optimisation, unlike our group's earlier experience with Human papillomavirus type 16 (HPV-16) L1 protein expression [52], as in that work we found that determination of best intracellular locale for transient protein accumulation was strictly empirical. Thus, while Pr55Gag accumulated similarly poorly in all locations, and p24 accumulated negligibly in cytoplasm and significantly better in both the ER and chloroplasts, p17/p24 yield was similar in cytoplasm and ER and 20-fold better in chloroplasts. Transgenic expression presented a different picture: cytoplasmic targeting of Pr55Gag resulted in regeneration failure, indicating toxicity; ER-targeted p24 did not accumulate well, although chloroplast targeting was equivalent to transient expression, and p17/p24 again accumulated best in chloroplasts, but to 20-fold lower levels than in transient studies.

We then investigated the feasibility of expressing HIV-1 p17/24 – a truncated Gag, but a more appropriate vaccine candidate than p24 – in *Nicotiana spp*. at levels suitable for processing for vaccine manufacture. We showed that transiently expressing the transgene under the CaMV 35S promoter produced the highest protein yields, up to 4 mg/kg (~0.3% TSP). The protein seemed to be relatively stable in these studies; however, results from transgenic plants showed higher recombinant protein yields in the young leaves than in the older, suggesting active turnover in the leaves rather than passive accumulation – in contrast to results for production of HPV-16 L1 protein [52]. The result clearly demonstrated that transient expression, and in particular, targeting the protein of a nuclear gene to the chloroplast, results in the best yield of recombinant p17/p24.

The protein yields reported in this study were below the expected economic threshold (1%). However, our laboratory has shown that up to 800 mg of HPV-16 L1 protein per kg fresh weight (12–17% total soluble protein) can accumulate in the chloroplasts of tobacco plants [52], meaning there is potential for much higher level accumulation.

Although results from electron microscopy were inconclusive it could be speculated that the HIV-1 p17/24 protein might form stable structures and possibly virus-like particles (VLPs), or tubules as reported by [41]. The myristylation site on the native Pr55Gag protein is essential for targeting the HIV-1 Pr55Gag protein to the plasma membrane: it is possible that myristylated p17/24 associates with the chloroplast membranes to make higher-order structures, and that non-membrane associated protein is toxic, which could account very well for the drastic difference in yield of the native and non-myristylated versions of p17/p24 (Figure 2). It is also possible that the membrane-associated form could be useful as a carrier for epitope presentation through C-terminal fusions, as is the full Pr55Gag protein made in insect cells [29]. In any case, further investigations are needed to characterise the nature of the structure(s) formed from the protein described here, and also its immunogenicity, if it is to be used as a candidate HIV-1 vaccine.

This study focussed on the immune response of mice to the leaf protein containing p17/p24 protein and was set up as proof of concept that plant-produced HIV proteins could possibly be further developed for candidate vaccines. As several studies have indicated enhanced immune responses can be achieved by heterologous prime-boost inoculation regimens, we chose to investigate the immune response of p17/p24 alone and its ability to boost an immune response to a DNA vaccine pTHGagC that is known to prime an immune response of mice, which can be effectively boosted by Pr55Gag VLPs [30]. Indeed, the substantial boost by leaf protein containing p17/p24 protein of Gag-specific cellular and humoral immune responses induced by the DNA vaccine pTHGagC suggests that HIV proteins produced in plants may be very valuable for expanding immune responses after the immune system has been primed. While both CD8+ and CD4+ vaccine-specific T cells have a protective role, CD4+ T cells are potentially important for the induction and maintenance of CD8 memory cells and stimulation of B cells for antibody production [53-55]. We found the increase in Gag-specific CD4+ T cells induced by the boost with p17/p24 protein is associated with a substantial increase in the Gag-specific humoral responses and a progression in an IgG1 to IgG2a isotype switch. Even though we did not measure Gag-specific Th1–Th2 responses, the IgG isotypes suggest our prime-boost inoculation regimen induces a mixed Th1–Th2 response with low doses of p17/p24 inducing more of a Th1 response, which changes to a greater Th2 type response when higher doses of p17/p24 are used. Although the inoculation with p17/p24 protein alone was not immunogenic in mice, we may find if we use a higher dose with homologous boosts or adjuvant, that a Gag-specific immune response is induced. Results obtained using a classic western blot strip assay for HIV antibodies also showed a clear boosting of the DNA-induced response, apparently to a greater degree than obtained by DNA prime-DNA boost (see Figure 6).

Our results highlight the potential of transient expression in plants for the production of HIV-l vaccine-relevant proteins. Until recently, transient expression through *Agrobacterium*-mediated infiltration was generally used only to verify gene expression, and to validate small amounts of recombinant proteins. However, it is now considered a genuine protein expression strategy in its own right that can potentially yield large amounts of protein [7,13,56,57]. Additionally, the short production cycle can reduce costs considerably compared to the equivalent transgenic case. The use of post-transcriptional silencing suppressors such as p19 and NSs [18], as well as increased protein stability after targeting proteins to cellular organelles, can further enhance the expression level. Furthermore, technologies have been developed to extract and purify antigens produced in plants for use in multi-component vaccines or immunogenic presentation of epitopes on VLPs or virions [7,13,56].

While many studies have been done to establish the potential of transplastomics for recombinant protein production, information on the use of plastid targeting tags such as rbsc1-cTP in pharmaceutical protein production is limited. However, a possible advantage lies in the fact that translation of the targeted proteins occurs in the cytoplasm, and then the proteins are imported into chloroplasts: when they are inside the chloroplast, the targeted proteins are then folded correctly,, making them more appropriately immunogenic. Using transit peptide tags might therefore overcome the limitations of using transplastomics, which include product insolubility, a lack of post-translational modifications, less established chloroplast transformation protocols and biosafety concerns of the horizontal transfer of transgenes from chloroplasts of transplastomics to bacteria under lab conditions [58].

## Conclusion

We determined that it was feasible to produce vaccine-relevant HIV-1 subtype C Gag-derived proteins in plants of *Nicotiana *spp. by means of transient expression via recombinant *A tumefaciens*, rather than via rTMV or transgenic expression. We demonstrated the positive effects on protein yield of plant codon optimisation, specific intracellular targeting, and protein composition. In particular, a p17/p24 fusion protein accumulated to relatively high levels in chloroplasts, was relatively easily partially purified from plants, and could be used to significantly boost both cellular and humoral immune responses in mice previously injected with an HIV DNA vaccine. This is the most vaccine-relevant HIV protein so far expressed in plants, and could have considerable potential.

## Methods

### Origin of DNA sequences

The HIV-1 *gag *gene used in these studies was from the cloned South African HIV isolate Du422 which has been used as the basis for South African HIV-1 subtype C vaccines (European Collection of Cell Cultures provisional accession no. 01032114; GenBank accession. AF544010) [3,4]. It was codon optimised for human and *Nicotiana *spp. expression and synthesised by GENEART GmbH (Germany). The capsid protein gene *p24 *and the *p17/24 *protein gene used in this work were derived from the *Nicotiana *spp. codon-optimised n*gag *gene.

### Viral vector expression

The wt*gag *ORF was cloned into the TMV Geneware™ vector pBSG1057 (Large Scale Biology Corporation, Vacaville, Ca., USA) at *Pac*I and *Xho*I sites to yield the clone pBSGwtgag. The n*gag *version was also cloned into pBSG1057 to yield pBSGngag. A wild type p24 ORF (wt*p24*) derived by PCR from wt*gag *as well as *Nicotiana *spp. and human codon-optimised versions n*p24 *and h*p24 *were cloned into pBSG1057 at the same restriction enzyme sites to yield the clones pBSGwtp24, pBSGnp24 and pBSGhp24. Recombinant viral RNA was transcribed from the clones using the RiboMAX™ Large Scale RNA Production System (T7) (Promega, Madison). The RNA was rub-inoculated onto leaves of *N. benthamiana *plants with gloved hands and a pinch of Celite (Merck, Germany). Plants were grown at 22°C under a 16 h/8 h light/dark cycle, monitored daily for TMV symptoms and leaves sampled at 3 to 10 days post inoculation (dpi) to test for Gag or p24 expression. Samples for electron microscopy for the detection of Gag VLPs were prepared using crude plant sap – plants were crushed in a minimal volume of 1 × PBS and the filtered extract clarified by centrifugation – from *N. benthamiana *plants infected with pBSGwtgag and pBSGngag RNA (10 dpi). Twenty microlitre amounts were immunotrapped onto carbon-coated copper grids (200 mesh) coated with HIV-1 p17 antiserum (ARP431, NIBSC). These were stained with 2% uranyl acetate and viewed under a Zeiss S1109 transmission electron microscope. Baculovirus-produced Gag VLPs were produced as previously described [30] for comparison.

### *Agrobacterium tumefaciens*-mediated transient expression

The n*gag*-derived *gag*, *p24 *and *p17/24 *ORFs were modified by PCR to attach the appropriate restriction enzymes sites to the 5' and 3'termini for cloning into the pTRA binary vectors: these are three pPAM-derived (Genbank Accession number AY027531) binary vectors, pTRAc, pTRAkc-ERH and pTRAkc-rbcs1-cTP [52], kindly provided by Dr Rainer Fischer (Fraunhofer Institute, Aachen, Germany). All three vectors have a cauliflower mosaic virus (CaMV) 35S promoter with a duplicated transcriptional enhancer, a CaMV 35S polyadenylation signal and a chalcone synthase 5' untranslated region for recombinant gene expression. Cloning into pTRAc results in cytoplasmic localisation; pTRAkc-ERH has a KDEL ER retention signal, while pTRAkc-rbcS1-cTP adds a chloroplast targeting signal from the small subunit of RUBISCO from *Solanum *(rbcS1-cTP). The HIV-1 *p24*, *p17/p24 *and *gag *genes were cloned into these vectors to yield 12 different constructs (Table 2). The gene encoding the p19 suppressor protein of Tomato bushy stunt virus (D Baulcombe, Sainsbury Lab, John Innes Centre, Norwich) was amplified by PCR with sense primer: 5' GGACGCGTTAGGTACATGGAACG AGCTATACAA 3', and antisense primer: 5' TCTAGACTCGAGTTACTCGCTTTC TTTTTCG 3', digested *Afl *III/*Xho *I and cloned into pTRAc, forming pTRAp19.

Two sets of clones were generated for the expression of HIV p17/24 genes in plants, one with the myristylation signal (p17/24-M) and the second lacking a myristylation signal (p17/24-NM). The attachment site of myristate occurs at the N terminal glycine [59] and this site was mutated from GGT (glycine) to GCT (alanine) by PCR. To generate appropriate constructs the p17/24 fusion sequence was amplified by PCR from pTHGagC. The fragments were cloned into the pTRA vectors to generate pTRA-Cp17/p24-M and pTRA-Cp17/p24-NM, pTRA-ERp17/p24-M and pTRA-ERp17/p24-NM, and pTRA-CPTp17/p24-M and pTRA-CPTp17/p24-NM respectively (Table 2).

Approximately 50 to 100 ng of recombinant plasmid was electroporated into host *A. tumefaciens *GV3101::pMP90RK cells as described by Maclean *et al*. [52] Recombinant *A. tumefaciens *colonies were selected on plates incubated at 27°C containing kanamycin (30 μg/ml), rifampicin (50 μg/ml) and carbenicillin (50 μg/ml).

For infiltration, recombinant strains were grown up overnight at 27°C with agitation in induction medium containing LB and 10 mM MES (pH5.6) supplemented with 20 μM acetosyringone, kanamycin (30 μg/ml), rifampicin (50 μg/ml) and carbenicillin (50 μg/ml). Cells were pelleted at 1000 g, resuspended in infiltration medium containing 10 mM MES and 1 M MgCl_2 _(pH 5.6) supplemented with 200 μM acetosyringone and diluted in infiltration medium to an OD_600 _of 0.25. After incubation at 22°C for 2 hours, cells were infiltrated by injection (1 ml syringe) or vacuum (-90 KPa for 1 min) into whole, uprooted *N. benthamiana *plants. Plants were co-infiltrated with *A. tumefaciens *LBA4404 (diluted in infiltration medium to an OD_600 _of 0.25) containing a silencing suppressor pBIN-NSs (provided by Marcel Prins, Laboratory of Virology, Wageningen, The Netherlands) which enhances transient protein expression by suppressing post transcriptional gene silencing [60]. This strain was similarly grown up overnight at 27°C with agitation in induction medium containing LB and 10 mM MES (pH 5.6) supplemented with 20 μM acetosyringone, kanamycin (30 μg/ml), rifampicin (50 μg/ml) and 2 mM MgSO_4 _to prevent the cells from clumping. Infiltrated plants were re-planted, grown at 22°C under a 16 h/8 h light/dark cycle and subsequently harvested at 3 dpi.

### Plant transformation

This work was carried out using 2–4 week-old *Nicotiana tabacum *cv. Petite Havana SRI plants grown in a greenhouse under a 16 hr light and 8 hr dark photoperiod at light intensity of 60–80 μE/m^2^/s and 22°C.

To generate transgenic plants, the recombinant *Agrobacterium *cultures containing the pTRA-CPTp17/p24-M and pTRA-ERp17/p24-M constructs were used to transform tobacco (*N. tabacum *cv. Petite Havana) by leaf disc transformation as described by [61]. Briefly, leaf discs (0.5 cm × 0.5 cm) were dipped in *Agrobacterium *overnight culture (OD_600 _= 0.6), regenerated under kanamycin selection (30 μg/ml) and rooted plantlets hardened off before being grown in a greenhouse under conditions described above.

### Immunoblotting

For western blotting, plant samples were ground in SDS-PAGE loading buffer and the proteins were separated by SDS-PAGE as described [52]. Proteins were transferred onto a nylon membrane and then challenged with rabbit polyclonal anti-p24 and goat anti-rabbit IgG (Sigma).

### ELISA analysis

For the plants infiltrated with p24, p17/24 and Pr55Gag constructs, six leaf discs (made using a 1.5 ml microfuge tube cap) per plant were harvested and ground up in 300 μl of extraction buffer (4 M urea, 100 mM DTT [18]) and liquid nitrogen. Samples were centrifuged for 10 min at 10000 rpm and the supernatant was used for ELISA with the appropriate sample dilutions. To detect the presence of HIV p17/24 a Vironostika^® ^HIV-1 antigen Microelisa kit (bioMérieux, Netherlands) was used. The kit is based on measuring HIV antigens using p24 murine monoclonal antibodies. For comparison, calculated antigen concentrations in the samples were normalised to the fresh leaf weight to obtain the yield of p24 per kg.

### Preparation of recombinant HIV-1 p17/p24-M

*A. tumefaciens *GV3101::pMP90RK cultures containing the pTRA-CTPp17/p24-M or pTRA-P19 clones were grown as described above. The *Agrobacterium *suspensions were diluted in infiltration medium to OD_600 _1.0, and were kept at 25°C for 2 h, then diluted and combined in infiltration medium, both to a final OD_600 _of 0.25. Whole *N. benthamiana *plants (3–4-weeks old) were vacuum-infiltrated: the plants were submerged into the bacterial suspension and subjected to a vacuum of -80 kPa for 2–5 min, with occasional agitation to release trapped air bubbles. The vacuum was released rapidly (~10 kPa/s). The plants were grown for 8 days under conditions of 16 h light, 8 h dark, 22°C.

Approximately 200 g of *N. benthamiana *leaves agroinfiltrated with pTRA-CPTp17/p24-M were harvested at 8 dpi and homogenised in a Waring blender in extraction bufffer (50 mM phosphate buffer containing 0.5 M NaCl, 5% glycerol, 0.05% Triton-X100, 1% sodium metabisulphite, pH 7.2). After 4 h incubation at 4°C the extract was centrifuged at 6 000 rpm for 20 min and the supernatant collected. PEG 6000 was added to 2% w/v, and the pH of the extract was adjusted to 7.2 with NaOH. The centrifugation was repeated and the supernatant was then concentrated 10-fold using a 10 kDa cut-off hollow-fibre column (GE Healthcare), and subjected to a 4 volume buffer exchange using a 50 mM phosphate buffer containing 0.5 M NaCl, 5% glycerol, 0.1 M ascorbic acid, at pH 7.2. Total leaf protein was determined to be 13 mg/ml and the p24 content determined to be 84 μg/ml by ELISA as described above.

The protein was assayed for purity by fractionation on 10% Coomassie-stained SDS-PA gels.

### pTHGagC and p17/p24 inoculum preparation

The DNA vaccine pTHGagC that expresses the Du422 HIV-1 subtype C Gag [4], was manufactured by Aldevron, Fargo, ND, USA and resuspended at 1 mg DNA/ml saline. Leaves infiltrated with p17/p24 were resuspended at 13 mg leaf protein/ml and determined to contain 84 μg p24/ml by a p24 ELISA assay (Vironostika^® ^HIV-1 antigen ELISA). A control with 13 mg leaf protein/ml was prepared from leaves that did not contain p17/p24.

### Immunization of BALB/c mice

Female BALB/c mice (8 – 10 weeks old) were divided into 8 groups with 5 mice per group. pTHGagC (100 μg DNA/100 μl saline) was given by injecting 50 μl into each tibialis anterior muscle. Leaf protein diluted in saline to give either 100 μg leaf protein/100 μl saline or 10 μg leaf protein/100 μl saline (containing the equivalent of 646 ng or 64 ng p17/p24 respectively) or control leaf protein (100 μg/100 μl saline) not containing p17/p24 was given intramuscularly by injecting 50 μl of the dose into each quadriceps muscle. pTHGagC was given as a primary inoculation to mouse groups 1 – 5 on day 0 while groups 6 – 8 were left unvaccinated until day 28. Group 1 received no further vaccination. On day 28 group 2 was given a booster injection of pTHGagC, groups 3 and 4 received either 646 ng or 64 ng p17/p24 protein and group 5 received 100 μg control leaf protein. 646 ng or 64 ng p17/p24 protein was given to groups 6 and 7 and group 8 received control leaf protein (100 μg) on day 28. All mouse groups were sacrificed on day 40.

### IFN-γ ELISPOT assay

A single cell suspension of splenocytes was prepared from spleens harvested on day 40 and pooled from 5 mice per group. IFN-γ ELISPOT responses were measured using a mouse IFN-γ ELISPOT set (BD Pharmingen). Splenocytes were plated in triplicate at 5 × 10^5^/well in a final volume of 200 μl R10 culture medium (RPMI with 10% heat inactivated FCS, Gibco, containing 15 mM β-mercaptoethanol, 100 U penicillin per ml, and 100 μg streptomycin). The peptides (>95% pure, Bachem, Switzerland) GagCD8 AMQMLKDTI and GagCD4 NPPIPVGRIYKRWIILGLNK were used as stimuli in the assay at a final concentration of 4μg/ml. Reactions containing an irrelevant H-2K^d ^binding peptide TYSTVASSL (obtained from Elizabeth Reap, AlphaVax) or without peptide served as background controls. Spots were detected with the detection antibody at 22 h, developed with Nova Red then counted using a CTL Analyzer (Cellular Technology, OH, USA) with Immunospot Version 3.0 software. The average number of spots in triplicate wells was calculated and results are expressed as the average number of spot-forming units (SFU) per 10^6 ^splenocytes ± the standard deviation (SD). For each group of mice the average background spots obtained in the absence of peptide and in the presence of the irrelevant peptide plus one standard deviation of this average was considered as the cut-off for a positive response.

### Antibody measurement by ELISA and western blot

To measure Gag specific antibodies in mouse serum, enzyme linked immunosorbent assay (ELISA) plates were coated with recombinant Gag protein (Quality Biologicals) at 1 μg/ml 0.1 M bicarbonate buffer (pH 9.5) overnight at 4°C. Plates were blocked with 10% FCS in PBS for 2 h at RT after which a 1:1000 dilution of serum samples pooled from 5 mice per group was added to the wells for a further 2 h at RT. A panel of goat-anti mouse antibodies specific for total IgG, IgG1, IgG2a, IgG2b and IgG3, obtained from Southern Biotechnology Associates (Birmingham, AL) as peroxidase-conjugated antibodies was used as per the manufacturer's instructions. Peroxidase substrate was reacted with *o*-phenylendiamine dihydrochloride (Sigma) and reactions at RT were stopped 10 min later with 2 N H_2_SO_4 _and the absorbance at 450 nm read on a Labsystems Multiskan Plus plate reader. The absorbance value for total IgG, IgG1, IgG2a, IgG2b and IgG3 with a 1:1000 dilution of serum taken prior to inoculation (pre-bleed value) was less than 0.033 ± 0.001 for all groups from which an absorbance value of 0.066 was decided as the cut off value for a positive response.

A commercial New LAV Blot I (BioRad) was used according to the manufacturer's instructions to determine the specificity of the antibodies in mouse serum. Mouse serum was used at a 1:40 dilution and antibody content detected with goat anti-mouse IgG conjugated to alkaline phosphatase.

### Statistical analysis

Statistical analysis was performed using the Student's *t *test.

## Authors' contributions

AM participated in the design of the study, made the full Pr55Gag and p24 targeted constructs, carried out the agroinfection and agroinfiltration experiments, analysed all the recombinant protein expression level data and drafted the manuscript. EC made the p17/p24 constructs and carried out the experiments associated with these. JM partially purified the protein required for the immunology studies. ES planned, supervised and carried out of the immunology studies. AL carried out the ELISAs and did some agroinfections. FLT cloned the wt, nicotiana and human optimised p24, transfected plants and helped to analyse data and draft the manuscript. A-LW and EPR participated in the coordination and design of the study, and drafted or helped to draft the manuscript. All authors read and approved the final manuscript.
